# Antiviral activity of betacyanins from red pitahaya (*Hylocereus polyrhizus*) and red spinach (*Amaranthus dubius*) against dengue virus type 2 (GenBank accession no. MH488959)

**DOI:** 10.1099/acmi.0.000073

**Published:** 2019-10-31

**Authors:** Ying Jun Chang, Lian Yih Pong, Sharifah S. Hassan, Wee Sim Choo

**Affiliations:** ^1^​ School of Science, Monash University Malaysia, Jalan Lagoon Selatan, 47500 Bandar Sunway, Selangor, Malaysia; ^2^​ Jeffrey Cheah School of Medicine and Health Sciences, Monash University Malaysia, Jalan Lagoon Selatan, 47500 Bandar Sunway, Selangor, Malaysia; ^3^​ Infectious Diseases and Health Cluster, Tropical Medicine and Biology Platform, Monash University Malaysia, Jalan Lagoon Selatan, 47500 Bandar Sunway, Selangor, Malaysia

**Keywords:** Betalain, cytotoxicity, viral infectivity, pathogenic, red dragon fruit, viral replication

## Abstract

This study investigated the antiviral activity of betacyanins from red pitahaya (*Hylocereus polyrhizus*) and red spinach (*Amaranthus dubius*) against dengue virus type 2 (DENV-2). The pulp of red pitahaya and the leaves of red spinach were extracted using methanol followed by sub-fractionation and Amberlite XAD16N column chromatography to obtain betacyanin fractions. The half maximum cytotoxicity concentration for betacyanin fractions from red pitahaya and red spinach on Vero cells were 4.346 and 2.287 mg ml^−1^, respectively. The half-maximal inhibitory concentration (IC_50_) of betacyanin fraction from red pitahaya was 125.8 μg ml^−1^ with selectivity index (SI) of 5.8. For betacyanin fraction from red spinach, the IC_50_ value was 14.62 µg ml^−1^ with SI of 28.51. Using the maximum non-toxic betacyanin concentration, direct virucidal effect against DENV-2 was obtained from betacyanin fraction from red pitahaya (IC_50_ of 126.70 μg ml^−1^; 95.0 % virus inhibition) and red spinach (IC_50_ value of 106.80 μg ml^−1^; 65.9 % of virus inhibition). Betacyanin fractions from red pitahaya and red spinach inhibited DENV-2 *in vitro*.

## Introduction

Dengue fever is an infectious disease caused by dengue virus (DENV). DENV is an arthropod-borne virus that is commonly distributed across the tropical and subtropical regions of the world and is transmitted to humans mainly by female *Aedes aegypti* mosquitoes [1]. There are four antigenically related, but serologically distinct types of DENV (DENV-1, 2, 3, 4), with DENV-2 being the most lethal serotype [2, 3]. Heterotypic secondary infection by a different serotype of DENV is thought to be associated with the elevated virus titre, due to antibody-dependent enhancement that can cause severe dengue diseases such as dengue haemorrhagic fever (DHF) or dengue shock syndrome (DSS) characterized by plasma leakage, thrombocytopenia, severe bleeding and can ultimately lead to organ failure and death [4]. Many studies focused on the control of DENV by combating mosquito vector, which is almost exclusively responsible for the transmission of DENV [5]. Currently, there is no antiviral therapy available for dengue infection and the only licensed dengue vaccine, namely Dengvaxia showed inconsistent efficacy across people [6]. The development of antiviral therapy is equally important in the control of dengue infection as well as preventing the progression of patient from dengue fever to DHF or DSS. Natural compounds as antiviral agents can be used in tandem with vaccine in controlling DENV infection.

Betacyanin, a red-violet pigment belongs to a class of plant pigment called betalain [7]. Betacyanin extracts from different sources of plant have a wide range of biological activities such as antioxidant, anticancer, antilipidemic and antimicrobial activities [8]. The antiviral activity of betacyanin was only reported against tobacco mosaic virus in plant [9], hence there is a research gap for the antiviral activity of betacyanin against human pathogenic viruses. Red pitahaya (*Hylocereus polyrhizus*) and red spinach (*Amaranthus dubius*) are two prolific crops commonly found in the tropical and sub-tropical countries where dengue is endemic. Both plants have multiple types of betacyanin and different betacyanin composition, hence they were selected as the sources of betacyanin. The objectives of this study were to investigate the cytotoxicity effect of betacyanin fractions from red pitahaya and red spinach on Vero cells, followed by investigating the antiviral activity of betacyanin fractions against dengue virus serotype 2 (DENV-2) via virus yield inhibition assay and virucidal assay. Only DENV-2 was investigated because it is the most lethal among four serotypes [3] and it has low efficacy for Dengvaxia vaccine especially for seronegative individuals [10].

## Methods

### ​Preparation and characterization of betacyanin fractions

Red pitahaya and red spinach were purchased from a supermarket (Bandar Sunway, Malaysia). Only fruits with 13–15 °Brix measured using a refractometer (Atago, Minato-ku, Japan) were used for the extraction to ensure the fruits were within the same range of ripeness. °Brix is a unit of measurement for total soluble solids and in this case, mainly sugar. This range of °Brix corresponds to a suitable degree of ripeness of the fruits as sugar content is correlated with degree of ripeness. Leaves of the red spinach with similar length (7–9 cm) were picked, washed and spin in a salad spinner to dry. This range of length is the typical length of the red spinach leaves. The pulp of red pitahaya and the red part of the leaves of red spinach were used for extraction. The sub-fractionated extracts were prepared according to a previous study [11]. The dried red pitahaya and red spinach crude extracts were dissolved in 100 ml distilled water and were adjusted to pH 2.0 by HCl. The extracts were then partitioned with equal volume of ethyl acetate. This step was repeated three times. The aqueous phase was collected and concentrated with the addition of ethanol by evaporation under low pressure at 40 °C. The sub-fractionated extracts were freeze dried and semi-purified using polyaromatic adsorbent resin Amberlite XAD16N (Sigma-Aldrich, St. Louis, USA). Briefly, the resin was soaked overnight in distilled water at 4 °C. Before packing into a glass column, the resin was rinsed with distilled water for three–four times until the supernatant was clear. Acidified water was prepared by adding trifluoroacetic acid to distilled water until pH 3 was reached. The packed column was rinsed with 1 l of distilled water and activated with 0.5 l of 2 % sodium hydroxide solution. The resin was then neutralized by rinsing with 0.5 l of distilled water, followed by conditioning to pH 3 with acidified water. The dried sub-fractionated red pitahaya (5.0 g) or red spinach (0.25 g) extracts was dissolved in 10 ml of distilled water, followed by loading into the column containing Amberlite XAD16N. The resin was then desalted by rinsing with 0.5 l of acidified water at a flow rate of 10 ml min^−1^ before eluting with acidified methanol (95 : 5, methanol: acidified water). The semi-purified extracts were then concentrated using rotary-evaporator at 40 °C, freeze dried and stored at −80 °C until analyses. These extracts were termed betacyanin fractions. The betacyanin content for dried red pitahaya and red spinach fractions were calculated [12] and HPLC analysis was carried out [13]. Agilent 6520 Accurate Mass Q-TOF LC/MS (Agilent Technologies, Santa Clara, USA) with dual ESI source operating in a positive ionization mode were used to perform liquid chromatography (LC)-MS/MS analysis [14].

### ​Host cell cytotoxicity assay

Vero cells (African green monkey kidney; ATCC CCL-18) were seeded on 96-well plates at 1×10^4^ cells ml^−1^ 1 day prior to conducting the assay. The confluent cell were treated with growth medium [1×minimum essential medium (MEM) containing 2 % fetal bovine serum (FBS), 1 % HEPES and 1 % penicillin-streptomycin antibiotic] containing various concentrations of betacyanin fractions from red pitahaya and red spinach for 48 h at 37 °C in a humidified incubator with 5 % CO_2_. The cells were washed once with PBS before adding 10 µl of 5 mg ml^−1^ of 3-(4,5-dimethylthiazol-2-yl)−2,5-diphenyltetrazolium bromide (MTT) reagent to each well containing 90 µl of PBS and incubated at 37 °C, 5 % CO_2_ for 4 h. Then 100 µl of solubilization solution (10 % SDS in 0.01 N HCl) was added and plate was further incubated overnight. The absorbance was measured using a microplate reader (Bio-Rad, Hercules, USA) at 560 nm (OD_560_). The cell viability (%) was calculated and the half maximum cytotoxicity concentration (CC_50_) was deduced from the dose-response curve.

### ​Virus yield inhibition assay

Plaque reduction neutralisation test (PRNT) was first carried out to determine virus yield inhibition. DENV-2 was isolated from a dengue-infected patient serum and confirmed by whole genome sequencing (GenBank accession no. MH488959). Vero cells were seeded on a 96-well plate and incubated at 37 °C with 5 % CO_2_ until 90–95 % confluency was reached. The cells were then infected with DENV-2 at an m.o.i. of 0.5 [15] for 1 h at 37 °C with 5 % CO_2_. Various concentrations of betacyanin fractions from red pitahaya and red spinach were prepared using milli-Q water. After 1 h of infection, the inoculum was removed and cells were washed once with PBS before incubating with growth medium containing diluted extracts for 48 h at 37 °C with 5 % CO_2_. A virus control consisting of infected cells without the addition of betacyanin fraction and a cell control were included as assay controls. The supernatants of infected cells were harvested and subjected to focus formation assay in duplicate after 48 h of post-infection. Focus forming assay [16] with some modifications was performed to allow quantification of the virus yield. Vero cells were seeded on 24-well plates at 5×10^4^ cells ml^−1^ a day before the assay. The cells were inoculated with 100 µl of supernatant, which was serially diluted in tenfold, and incubated at 37 °C with 5 % CO_2_ for 1 h. The plate was gently rocked every 12 min during 1 h of infection. The inoculum was then removed and cells were overlaid with 2 % carboxymethyl cellulose (CMC) overlay medium, followed by incubating at 37 °C with 5 % CO_2_ for 4 days. The overlay medium was removed and cells were washed twice with 1×Tris-buffered saline (TBS) containing 0.1 % Tween 20 (TBST). The cells were then fixed with cold 80 % acetone for 10 min at room temperature followed by washing. After the incubation with blocking buffer [1 % bovine serum albumin (BSA), 0.5 % Triton X-100 in TBST] at 37 °C with 5 % CO_2_ for 45 min, the cells were incubated with mouse monoclonal dengue virus type 1, 2, 3 and 4 antibody [D1-11(3)] (GeneTex) at 37 °C for 1 h. After three times of washing for 5 min, the cells were incubated with alkaline phosphatase-conjugated goat antimouse IgG (GeneTex) at 37 °C for 1 h followed by washing. The cells were then incubated with a mixture of NBT (nitrotetrazolium blue chloride) and BCIP (5-bromo-4-chloro-3’-indolyphosphate p-toluidine salt) substrates (Bio Basic) for 10 min. The immunostained plates were rinsed under running water and allowed to dry before counting the foci. The percentage of focus inhibition was then calculated. The half-maximal inhibitory concentration, IC_50_, was determined as the concentration of betacyanin fraction, which causes 50 % reduction in virus yield.

### ​Virucidal assay

Vero cells were seeded on 24-well plates at 5×10^4^ cells ml^−1^ a day before virucidal assay. In a 96-well plate, various concentrations of betacyanin fractions prepared in milli-Q water were incubated with 30 foci-forming units (f.f.u.) of DENV-2 at an equal volume for 1 h at 37 °C with 5 % CO_2_ before being transferred onto cells in the 24-well plate. FFA was then performed as described above. The IC_50_ values were determined as mentioned above.

### ​Statistical analysis

GraphPad Prism software (version 7.04) was used for the statistical analyses, determination of CC_50_ and IC_50_, and graphical illustrations of antiviral effects of betacyanin fractions from red pitahaya and red spinach. The virus yield inhibition assay and virucidal assay were performed in duplicate (two technical replicates) using two independent red pitahaya and red spinach extracts (two independent extraction).

## Results and discussion

Betalains are water-soluble nitrogen-containing pigments, which can exist as the red-violet betacyanins or yellow betaxanthins. The aglycon betanidin is the backbone of all betacyanins in which the occurrence of different betacyanin structures is due to the glycosylation and acylation of the 5-*O*- or 6-*O*-glucosides [7]. The most abundant betacyanin found in betacyanin fraction from red pitahaya was phyllocactin, followed by betanin, hylocerenin and isobetanin (Table 1). In betacyanin fraction from red spinach, the most abundant betacyanin found was amaranthine, followed by decarboxy-amaranthine and betanin. The betacyanin composition of betacyanin fractions from red pitahaya and red spinach in this study is similar to a previous study [13]. The purity of betacyanins in these fractions were higher than those of previous studies [11, 13] as column chromatography was employed to further purify the extracts from red pitahaya and red spinach. The bioactivity of the betacyanin in these fractions can only be confirmed by isolating individual betacyanin and determine its bioactivity to prove that any effect is really due to the predominant betacyanins present and not due to some minor constituents. Amaranthine was previously isolated from red spinach extract using preparative HPLC and proven to possess antibiofilm activity similar to its extract [11].

**Table 1.** Betacyanins characterized by HPLC and LC-MS/MS in betacyanin fractions from red pitahaya and red spinach

Compound	Molecular weight (g mol^−1^)	[M+H]^+^ m/z	MS^2^ m/z	Percentage (%)
**Red pitahaya**				
Betanin	550	551	389	28.98±0.62
Isobetanin	550	551	390	5.57±0.47
Phyllocactin	636	637	594, 390	51.3±0.12
Hylocerenin	695	696	598, 387	14.12±0.69
**Red spinach**				
Amaranthine	726	727	679, 540, 390	70.27±0.11
Decarboxy-amaranthine	682	683	594, 508 346	21.57±0.29
Betanin	550	551	389	8.15±0.26

**Fig. 1.**
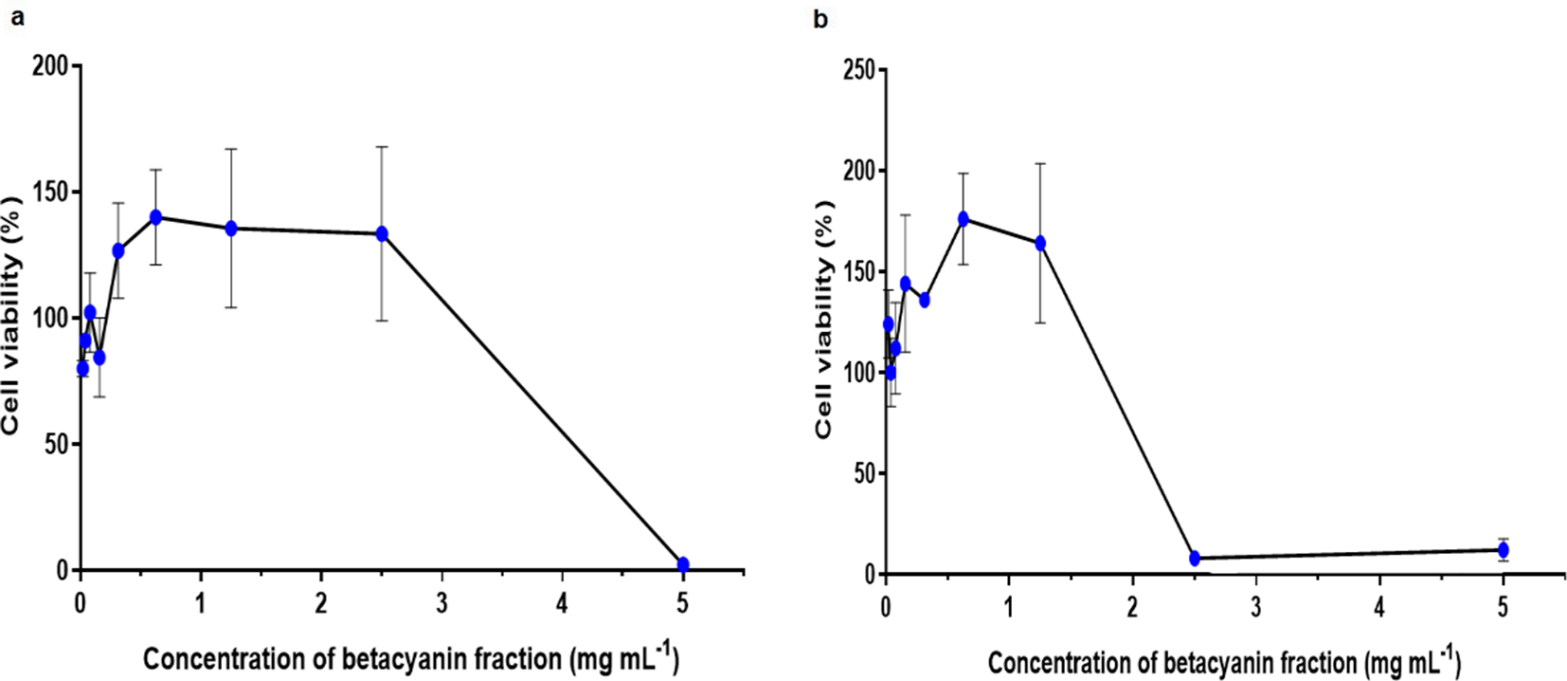
Cytotoxicity effect of betacyanin fraction from (a) red pitahaya and (b) red spinach on Vero cells. Values represent the mean±sem of assay performed in duplicate. The half maximum cytotoxicity concentration (CC_50_) values were obtained using GraphPad Prism software.

The half maximum cytotoxicity concentration (CC_50_), which is the concentration required to reduce cell viability by 50%, was at 4.38 mg ml^−1^ (betacyanin content: 664.73 μg ml^−1^) for betacyanin fraction from red pitahaya (Fig. 1a) and at 2.42 mg ml^−1^ (betacyanin content: 416.83 μg ml^−1^) for betacyanin fraction from red spinach (Fig. 1b). The betacyanin fractions from red pitahaya and red spinach were considered to be non-cytotoxic to Vero cells at the extract concentrations below 2.50 mg ml^−1^ (betacyanin content: 379.50 μg ml^−1^) and 1.25 mg ml^−1^ (betacyanin content: 215.75 µg ml^−1^), respectively. The antiviral assays were then conducted using these ranges of non-cytotoxic concentrations. There was an increase in the cell viability at the non-toxic concentrations. This may be due to hormesis in the Vero cells whereby a dose-response relationship is exhibited showing low-dose stimulation and high-dose inhibition [17]. In addition, the large error bars at these concentrations could be reduced with larger replicates. There was only one study reporting on the cytotoxicity effect of betacyanin extract from red pitahaya on human embryonic kidney (HEK-293) cells and human monocytes (THP-1) [18]. This study is the first to report of the cytotoxicity of betacyanin fractions from red pitahaya and red spinach on Vero cells. It was suggested that a daily intake of not more than 100 mg of betanin (a type of betacyanin) in purified form can be used as colourants and providing effective bioactivity [19].

**Fig. 2.**
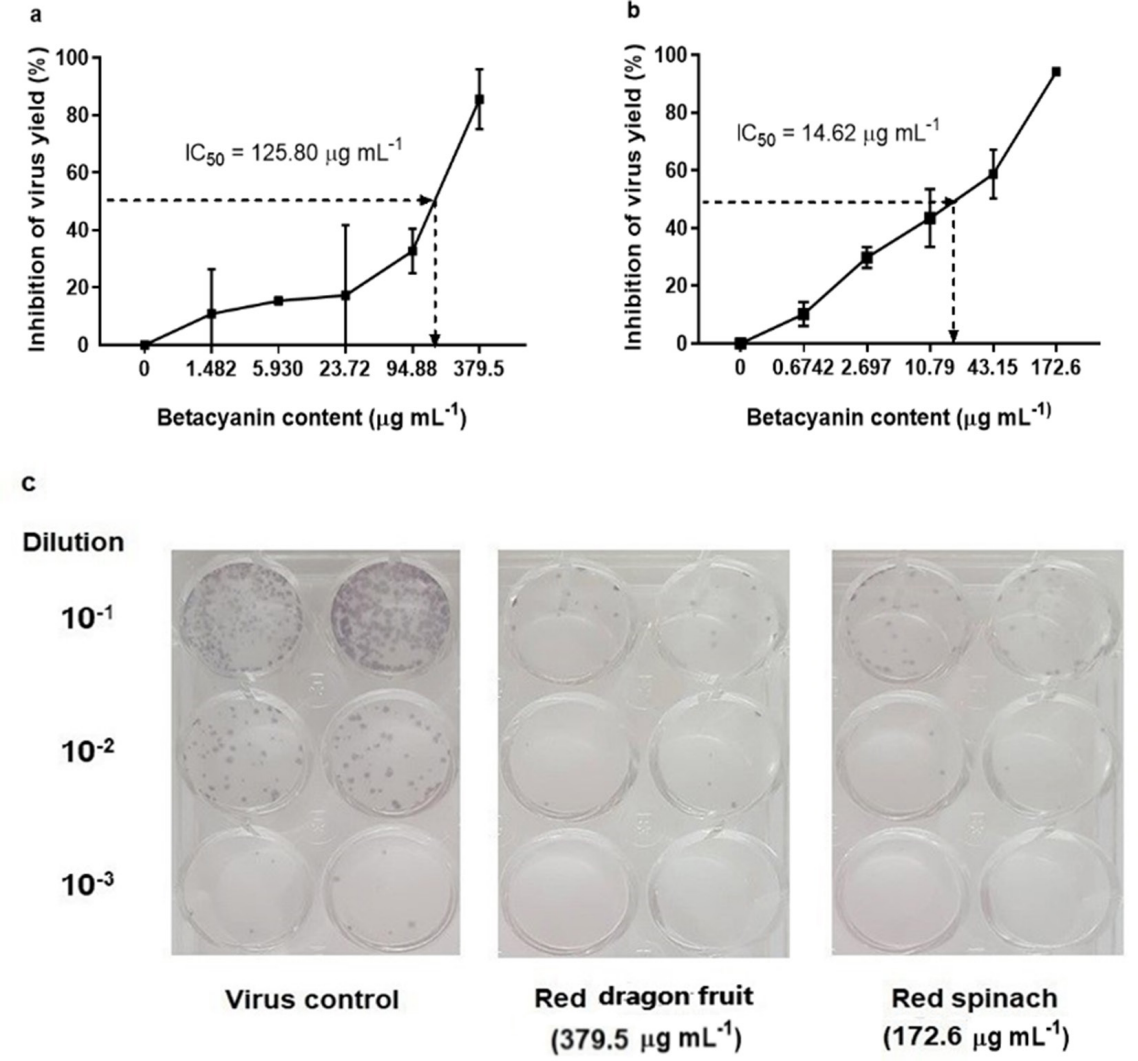
Antiviral effect of betacyanin fractions from (a) red pitahaya and (b) red spinach. The IC_50_ values were obtained using GraphPad Prism software. (c) The representative focus formation of DENV-2 in Vero cells treated with betacyanin fractions from red pitahaya and red spinach at the maximum non-toxic betacyanin concentration.

**Fig. 3.**
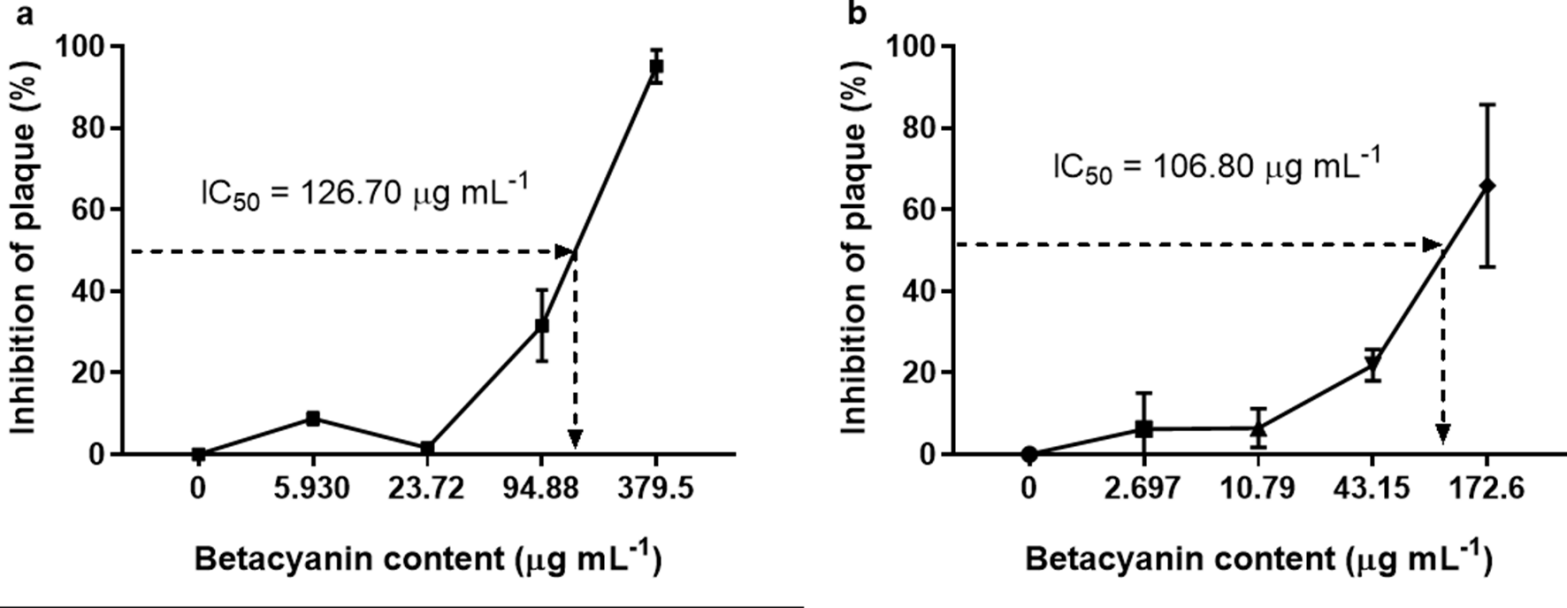
Virucidal effect of betacyanin fractions from red pitahaya and red spinach. The dose-response of plaque inhibition (%) against betacyanin content (μg ml^−1^) of betacyanin fraction from (a) red pitahaya and (b) red spinach against DENV-2. The IC_50_ values were obtained using GraphPad Prism software.

Betacyanin fractions from red pitahaya and red spinach exhibited antiviral activity against DENV-2 after virus adsorption to the host cells in a dose-dependent manner (Fig. 2). The IC_50_ of betacyanin fraction from red pitahaya was 125.8 μg ml^−1^, with selectivity index (SI) of 5.28. SI was calculated by dividing CC_50_ over IC_50_. For betacyanin fractions from red spinach, the IC_50_ value was 14.62 μg ml^−1^, with a SI of 28.51. These results suggest that the infectivity of DENV-2 was suppressed by the betacyanins from red pitahaya and red spinach. However, further investigation on measuring the cellular processes of DENV-2 affected by incubation with betacyanins should be carried out in the future to determine whether betacyanins interfere with the cellular processes used by the virus in its life cycle. To evaluate the virucidal effect of betacyanin fractions on DENV-2, various non-toxic concentrations of betacyanin fraction were incubated with DENV-2 directly and the virus infectivity was assessed via focus formation assay. Betacyanin fraction from red pitahaya demonstrated direct virucidal effect against DENV-2 with an IC_50_ of 126.70 μg ml^−1^ and with 95.0 % of virus inhibition (Fig. 3a) at the maximum non-toxic betacyanin concentration (379.5 μg ml^−1^). As for betacyanin fraction from red spinach, the IC_50_ value was 106.8 μg ml^−1^ and the virus inhibition was 65.9 % (Fig. 3b) at the maximum non-toxic betacyanin concentration (172.6 μg ml^−1^). These results of virucidal assay imply that betacyanin fractions from red pitahaya and red spinach may be able to inactivate the extracellular DENV-2 particles. The betacyanins most probably bind to the virus particles, thus inactivating the DENV-2 from initiating the virus infection. Previous studies showed that natural compounds, which can inhibit DENV replication show interactions with the non-structural proteins [20, 21]. Thus, it is deemed that betacyanin might interact with the non-structural protein especially the envelope (E) protein of DENV-2, which is crucial for virus attachment, suppressing the infectivity of DENV-2 by interfering the attachment of virus to the host cell [22, 23]. However, the mechanisms of inhibition by the betacyanins on DENV-2 need to be confirmed by further investigation.

For both dose-dependent antiviral assay and virucidal assay, betacyanin fraction from red spinach seem to have a better antiviral effect against DENV-2 than red pitahaya, whereby the IC_50_ values of betacyanin fraction from red spinach were lower than those of betacyanin fraction from red pitahaya. In addition, the higher SI value of betacyanin fraction from red spinach than that of red pitahaya also suggests the better antiviral activity of betacyanin fraction from red spinach. Individual betacyanin should be isolated to identify the candidate exerting the highest antiviral effect. The IC_50_ values of betacyanin fraction from red spinach (14.6 μg ml^−1^) for the inhibition DENV-2 is also lower when compared with some other natural compounds such as baicalein rich *Scutellaria baicalensis* extract (86.59 to 95.19 μg ml^−1^) and quercetin (28.90 μg ml^−1^) [24, 25], indicating a better antiviral property of red spinach. Both compounds belong to the flavonoid group of polyphenols. The findings in this study suggest that the betacyanin fractions from red pitahaya and red spinach can be potentially to be developed as antiviral agents as part of antiviral therapy against DENV-2. To further investigate the effectiveness of these betacyanin fractions in inhibiting the DENV infection, the determination of mode of action of these extracts and *in vivo* study are needed to be carried out in future.
